# Severe hypersensitivity reactions to iodinated contrast media: clinical and immunological features in a cohort of patients

**DOI:** 10.1186/2045-7022-4-S3-P131

**Published:** 2014-07-18

**Authors:** Daniela Rivero Paparoni, Ana Fiandor, Rosario Cabañas, Hoi Yan-Tong, Elena Ramirez, Marta Oñate, Santiago Quirce

**Affiliations:** 1 Department of Allergology, Hospital La Paz, Health Research Institute (IdiPAZ), Spain; 2 Hospital La Paz. Health Research Institute (IdiPAZ), Allergy, Spain; 3 Hospital La Paz. Health Research Institute (IdiPAZ), Clinical Farmacology, Spain; 4 Hospital La Paz. Health Research Institute (IdiPAZ), Radiology, Spain

## Background

Iodinated contrast media (ICM) are diagnostic agents used in millions of X-ray procedures yearly. Although current agents are usually well tolerated, mild immediate hypersensitivity reactions (IHR) occur in about 0.15% to 3% of administrations and severe IHRs in 0.01% to 0.04%. Despite this low incidence these reactions are an important clinical problem because the increasingly large amount of administrations per year worldwide. New data indicate that many of the immediate reactions are caused by an IgE-mediated activation of mast cells in the organs affected.

## Methods

A retrospective review of adverse reactions to ICM between 2011 and 2012 in a tertiary hospital was carried out. Allergy study was performed in all patients who developed immediate hypersensitivity reactions. Skin tests for contrast media (Iomeprol, Iobitridol, Iohexol, Iodixanol, Amidotrizoato) were performed. We analysed the characteristics of patients who had severe reactions (Grade III-IV de Müller).

## Results

73,962 radiological contrast studies were performed during 2011 and 2012. 80 (0.011%) patients showed immediate hypersensitivity reactions to contrast media, 4 of them had severe reactions to ICM. Three patients, 2 males and 1 female, ages ranging from 60 to 78 years, were studied in Allergy unit. One patient had atopy. Two patients were on antihypertensive treatment (beta-blockers, angiotensin II receptor blockers, calcium channel blockers). All patients had at least 1 previous study with iodinated contrast in the past that had been well tolerated. Iomeprol was the contrast media involved in all the patients, incidence of 0.008% (Poisson 95% CI: 0.004% to 0.015%), no fatality. Skin tests (Skin Prick Test in one patient, and Intradermal test on the other two) were positive for Iomeprol. 2/3 showed negativity for the other contrasts tested and 1/3 showed also a positive response to Iohexol. Both positives results were confirmed with basophil activation test.

## Conclusion

Incidence of severe immediate reactions to iodinated contrast media is very low. They are IgE mediated at least in 75% of cases in our cohort. There was at least one previous exposure to iodinated contrast before developing the reaction.

